# Isolation, characterization and therapeutic evaluation of a new Acinetobacter virus Abgy202141 lysing *Acinetobacter baumannii*

**DOI:** 10.3389/fmicb.2024.1379400

**Published:** 2024-04-30

**Authors:** Xun Tian, Xiang Liu, Jianhong Zhou, Li Wang, Qinrong Wang, Xiaolan Qi, Jiayu Liu, Dailin Zhao, Tom Hsiang, Yinhui Jiang

**Affiliations:** ^1^Key Laboratory of Endemic and Ethnic Diseases, Guizhou Medical University, Ministry of Education, Guiyang, China; ^2^Key Laboratory of Medical Molecular Biology, Guizhou Medical University, Guiyang, Guizhou, China; ^3^Guizhou Provincial Center for Clinical Laboratory, Guiyang, China; ^4^Key Laboratory of Medical Insects, Guizhou Medical University, Guiyang, Guizhou, China; ^5^Institute of Plant Protection, Guizhou Academy of Agricultural Sciences, Guiyang, China; ^6^School of Environmental Sciences, University of Guelph, Guelph, ON, Canada

**Keywords:** *Acinetobacter baumannii*, phage, genome analysis, biological characteristics, phage therapy

## Abstract

*Acinetobacter baumannii* is an opportunistic pathogen that easily resists currently available antibiotics. Phages are considered alternative therapeutic agents to conventional antibiotics for the treatment of multidrug-resistant bacteria. We isolated an Acinetobacter virus Abgy202141 from underground sewage in a residential area of Guiyang City in China. Transmission electron microscopy (TEM) analysis showed that Acinetobacter virus Abgy202141 has an icosahedral head attached to a tail. This phage infects *A. baumannii* strain GY-4, and was found to have a short latent period of 5 min and with a burst size of 189 particles per infected host cell. Additionally, Acinetobacter virus Abgy202141 remained stable at different concentrations of chloroform and varying pH levels and temperatures. Based on SDS-PAGE analysis, it contained 14 proteins with molecular weights ranging from 12 to 125 kDa. The double-strand (ds) DNA genome of Acinetobacter virus Abgy202141 consisted of 41,242 bp with a GC content of 39.4%. It contained 50 open reading frames (ORFs), of which 29 ORFs had identified functions, but no virulence-related genes, antibiotic-resistance genes, or tRNAs were found. Phylogenetic analysis indicated that Acinetobacter virus Abgy202141 was a new phage in the *Friunavirus* genus. Acinetobacter virus Abgy202141 also showed the ability to prevent *A. baumannii* infections in the *Galleria mellonella in vivo* model.

## 1 Introduction

*Acinetobacter baumannii* is a gram-negative bacterium that is commonly found in hospital wound infections (Micelli et al., 2023). Its ability to produce biofilms and broad resistance to antibiotics make it one of the most successful opportunistic pathogens in the hospital environment (Altnok et al., 2020; Anane et al., 2020). As reported, over 80% of *A. baumannii* isolates from intensive care units and other wards were resistant to most antibiotics in a general public hospital of Greece (Feretzakis et al., 2019). An infection of *A. baumannii* may lead to bacteremia, ventilator-associated pneumonia, and urinary tract infections (Altnok et al., 2020).

Numerous antibiotic resistance processes have been found with *A. baumannii*, including enzyme inactivation, target modification, active efflux, and reduced chemical uptake (Rajkumari and Siddhardha, 2020). New or alternative management methods are needed. Phages are abundant in the environment, and they can specifically attack pathogenic bacteria (Tu et al., 2023). Phages may be found that can attack Multidrug-Resistant (MDR) strains of *A. baumannii* which may be a potential solution for this pathogen (Tu et al., 2023).

In this study, we isolated and identified a new Acinetobacter virus Abgy202141 infecting *A. baumannii*. The genome of the phage was sequenced and its characteristics were analyzed. The therapeutic use of phage combined with antibiotics was evaluated in the *G. mellonella* model. The results provide more options to treat with antibiotic resistance in *A. baumannii*.

## 2 Materials and methods

### 2.1 Bacterial strains and culture conditions

*A. baumannii* strain GY-4 was isolated from samples from infected patients at Guizhou Medical University Affiliated Hospital, Guiyang, Guizhou. The strain is resistant to beta-lactamase antibiotics and carries the TEM beta-lactamase (*blaTEM*) gene (Chen et al., 2016). The strain was grown in Luria-Bertani (LB) liquid medium (tryptone 10 g/L, yeast extract 5 g/L, and NaCl 10 g/L) or plated onto solid LB medium containing 1.5% (w/v) agar and cultured at 37°C (Wu et al., 2018).

### 2.2 Phage isolation and purification

Isolation and purification of the Acinetobacter virus Abgy202141 were performed as described previously with slight modifications (Zhang et al., 2017; Yang et al., 2019). *A. baumannii* strain GY-4 was used as a host for the isolation of phages from underground sewage, which had been treated by means of several steps of filtration by the wastewater treatment plant of Guiyang City. However, the treatment of underground sewage, i.e. the baiting process, was performed in a biosafety cabinet under Biosafety Level 2 (BSL-2) conditions, and infected hosts were retrieved under those conditions. Approximate 250 mL sewage was treated with CaCl_2_ at a final concentration of 1 mmol/L and incubated for 10 min at room temperature. The treated sewage was centrifuged at 8000 rpm at 4°C for 10 min and passed through 0.22 μm pore-sized filters to obtain the supernatant. And then, 50 mL of LB and 1 mL of GY-4 culture at the exponential phase were added to the supernatant. Then the mixture was incubated overnight at 37°C with shaking at 200 rpm. The treated mixture was centrifuged at 8000 rpm at 4°C for 10 min and passed through 0.22 μm pore-sized filters to obtain the supernatant. The supernatant (0.1 mL) was mixed with exponentially growing bacteria (0.1 mL), and incubated at room temperature for 15 min. The mixture was added to 5 mL of LB soft agar overlay (0.75% agar), mixed briefly, and spread over an LB plate. Plates were incubated at 37°C overnight to obtain the single phage plaques. The phage was purified at least three times with the single phage plaque technique. The last supernatant from the culture was filtered through 0.22 μm pore-sized filters and stored at −80°C.

### 2.3 The double-agar plaque assay

The double-agar plaque assay was performed as described previously with slight modifications (Wu et al., 2018; Hao et al., 2023). Single plaque was selected from a double-layer plate and added to exponentially growing bacteria (10 mL). Then the mixture was incubated at 37°C with shaking at 200 rpm until the solution was clear. The treated mixture was centrifuged at 8000 rpm at 4°C for 10 min and passed through 0.22 μm pore-sized filters to obtain the phage fluid. The phage fluid was diluted by 10 fold gradient. The phage fluid of appropriate dilution (0.1 mL) was mixed with exponentially growing bacteria (0.1 mL) and incubated at room temperature for 15 min. The mixture was added to 5 mL of LB soft agar overlay (0.75% agar), mixed briefly, and spread over an LB plate. Plates were incubated at 37°C overnight to obtain the single phage plaque. Phage titer (PFU/mL) was calculated as the number of plaques × dilution factor × 10.

### 2.4 Transmission electron microscopy

The morphological characteristics of Acinetobacter virus Abgy202141 were examined as described previously with slight modifications (Yuan et al., 2021). The purified phage suspension (about 10^12^ PFU/mL) was stained with 2% (w/v) uranyl acetate. The morphological characteristics of the phage were observed using a transmission electron microscope (TEM, Hitachi H-7650, Tokyo, Japan) at 80 kV.

### 2.5 Multiplicity of infection

The multiplicity of infection (MOI) is the proportion of virus particles to host cells (Wei et al., 2023). A phage infection curve was assayed using the method described by Wu et al. (2018). The host cells of *A. baumannii* strain GY-4 were mixed with Acinetobacter virus Abgy202141 at various MOIs (0.00001, 0.0001, 0.001, 0.01, 0.1, and 1) incubated at 37°C, and shaking with 150 rpm for up to 6 h. The PBS buffer was used for the control. OD600 levels were measured at 30-minute intervals for 6 h.

### 2.6 One-step growth curve

The one-step growth curve of Acinetobacter virus Abgy202141 was evaluated as described previously with slight modifications (Pajunen et al., 2000; Wu et al., 2018). The host cells of *A. baumannii* strain GY-4 were infected with Acinetobacter virus Abgy202141 at the MOI of 1, and then incubated at 37°C with 150 rpm; and then the samples were collected at intervals (1, 2, 3, 4, 5, 6, 7, 8, 9, 10, 15, 20, 30, 40, 50, 60, 90, 120, 180, and 240 min). The double-agar plaque assay was used for phage titer after each interval of incubation to estimate titer.

### 2.7 Phage stability

The heat stability of Acinetobacter virus Abgy202141 was tested at different temperatures (4, 37, 45, 55, 65, or 75°C) for 1 h (Kropinski et al., 2009). The pH stability of Acinetobacter virus Abgy202141 was tested at different pH values (pH 2, 3, 4, 5, 6, 7, 8, 9, 10, 11, 12, and 13) for 1 h at 37°C (Cha et al., 2018; Wu et al., 2018). The double-agar plaque assay was used for each sample to estimate titer.

### 2.8 Chloroform sensitivity

The chloroform tolerance of phages is a crucial reference to determine the presence or absence of lipid components in the capsids or tail of phages (Wei et al., 2023). So the chloroform tolerance of the phage was assessed as described previously with slight modifications (Wei et al., 2023). Acinetobacter virus Abgy202141 preparation (100 μL) was mixed with 900 μL of chloroform at various concentrations (0, 1%, 3%, and 5%), and the double-agar plaque assay was used to determine the resulting phage titer.

### 2.9 DNA extraction, sequencing and genomic analysis

The phage DNA was extracted using the phenol-chloroform technique (Pickard, 2009). The extracted DNA was sent to Shenggong Biotechnology Co., Ltd (Shanghai) for sequencing and assembly. Briefly, a DNA library was obtained using the Illumina TruSeq™ Nano DNA Sample Prep Kit instructions. Sequencing was done on the Illumina NovaSeq sequencing platform with paired-end 150 bp reads. Low-quality reads were filtered out by Trimmomatic v0.36 (*Q*-value < 20, 98.51%). A5-MiSeq v20160825 and SPAdes v3.12.0 were used to assemble sequencing data to contigs and scaffolds. MUMmer v3.1 and Pilon v1.18 were used to fill the remaining inner local gaps and fix the single-base polymorphism for the final assembly.

ORFs of Acinetobacter virus Abgy202141 were found using RAST server (http://rast.nmpdr.org/rast.cgi). BLASTn (https://blast.ncbi.nlm.nih.gov/Blast.cgi?PROGRAM=blastn&PAGE_TYPE=BlastSearch&BLAST_SPEC=&LINK_LOC=blasttab&LAST_PAGE=blastn) was used to assess the similarity of phage genomes to data on GenBank. BLASTp was used to evaluate similarity to known proteins and obtain potential functions. Putative tRNA sequences were revealed using tRNAscan-SE (Chan and Lowe, 2019). The CDD database was used to search for conserved domains in phage sequences (Lu et al., 2020). The online databases VFDB (https://cge.cbs.dtu.dk/services/VirulenceFinder/) and CRDB (https://cge.cbs.dtu.dk/services/ResFinder/) were used to find potential virulence factors and antibiotic resistance genes (Alcock et al., 2020; Liu et al., 2022). The complete genomic map and GC offset of Acinetobacter virus Abgy202141 were created using CGview Server (http://stothard.afns.ualberta.ca/cgview_server/). The complete genome alignment map of Acinetobacter virus Abgy202141 was created using EasyFigure (2.2.5) (Sullivan et al., 2011). The phylogenetic tree was constructed by amino acid sequences of RNA polymerase with the Maximum Likelihood method (Tamura et al., 2011). And the optimal tree was statistically assessed with a bootstrap of 1,000-replicates.

### 2.10 SDS-PAGE analysis

Sodium dodecyl sulfate-polyacrylamide gel (SDS-PAGE) analysis of Acinetobacter virus Abgy202141 was performed as described previously with slight modifications (Yang et al., 2010). The purified particle suspension was loaded on a 12% SDS-PAGE gel. The protein bands were visible by staining the gel with Coomassie Blue Fast Staining and No-decoloring Solution (Epizyme, Shanghai, China).

### 2.11 *In vivo* synergy in the *G. mellonella* model

In order to remove endotoxins of *A. baumannii*, the phage suspension was prepared as described previously with slight modifications (Hietala et al., 2019). Briefly, 50 ml of Abgy2021-4-1 lysate was treated with 1 M NaCl for 1 h on ice, and then the solution was passed through 0.22 μm pore-sized filters. PEG 8000 was dissolved in the supernatant at a final concentration of 10% (m/v) and the solution was incubated at 4°C for 24 h. The treated solution was centrifuged at 4°C at 8000 rpm for 15 min and the pellet was dissolved in 1 ml of PBS buffer. The solution was extracted with chloroform (1 mL) twice, and the mixed solution was centrifuged at 4°C at 8000 rpm for 20 min. Finally, the phage suspension was obtained using 0.22 μm pore-sized filters.

The injections were performed as described previously (Grygorcewicz et al., 2020). *G. mellonella* larvae (~300 mg with cream color) were injected with 10 μL bacterial cells (*A. baumannii*) of different concentrations (10^3^, 10^4^, 10^5^, 10^6^ CFU/larva). Twenty minutes after bacterial cell injection, 10 μL purified phage particle suspensions with different MOIs (MOI = 50, MOI = 10, MOI = 1, and MOI = 0.1) were injected into the infected larvae. *A. baumannii* strain GY-4 is resistant to ampicillin (AMP), but sensitive to imipenem (IPM). Therefore, IPM injection was used as a positive control, and AMP injection was used as a negative control. The AMP or IPM treatments combined with phage was also used for treating GY-4 infection in *G. mellonella* larvae. Based on clinical doses, ~10 μL of antibiotics were injected into each larva at a final concentration of 18.75 mg/kg of AMP, or 50 mg/kg of IPM (Joly-Guillou et al., 1997; Grygorcewicz et al., 2020; Wang et al., 2021). For survival rate assays, the presence of dark-colored larvae with no response to physical contact within 120 h were recorded as dead (Joly-Guillou et al., 1997; Grygorcewicz et al., 2020; Wang et al., 2021). The control and check groups contained untreated larvae, pierced larvae, and PBS-injected larvae (PBS).

### 2.12 Statistical analysis

All experimental data were statistically analyzed and plotted with the software GraphPad Prism 8.0.2 and SPSS Statistics 21.0. The Kaplan-Meier method was used to plot the survival curve, and the log-rank test was used for survival curve analysis. *P* < 0.05 indicated statistically significant differences.

## 3 Results and discussion

### 3.1 Isolation and morphology of phage

**Figure 1:**
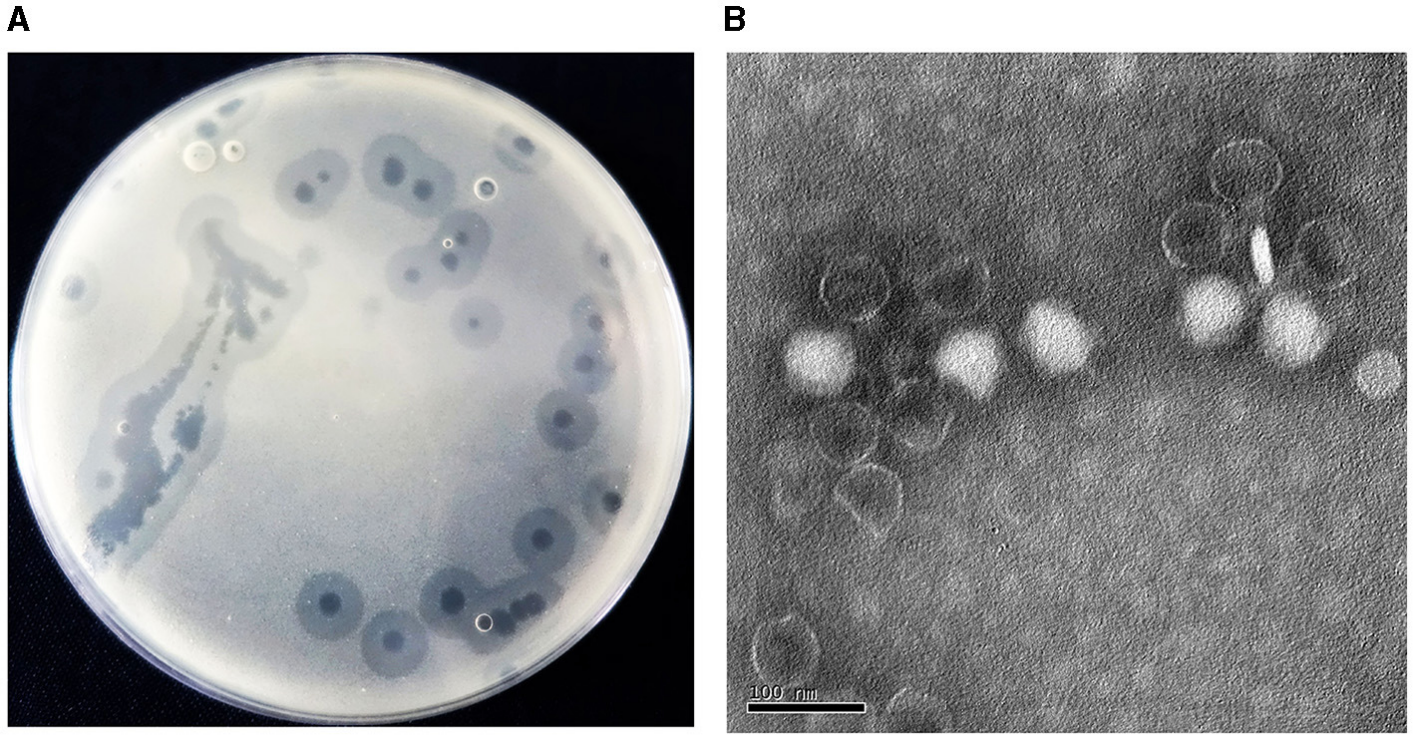
**(A)** Plaques formed by Acinetobacter virus Abgy202141 after 12 h incubation at 37°C. **(B)** Transmission electron micrograph of Acinetobacter virus Abgy202141. The bar indicates 20 nm.

Acinetobacter virus Abgy202141 directed against *A. baumannii* strain GY-4 was isolated from underground sewage from residential areas of Guiyang City. In the double-agar plaque assay, Abgy202141 could form transparent circular plaques of 1 cm diameter (Figure 1A). Transmission electron microscopy (TEM) analysis showed that Acinetobacter virus Abgy202141 had an icosahedron head (~59 nm) with a short tail (~23 nm) (Figure 1B). The underground sewage from residential areas contains abundant bacteria and phages, and it can serve as a preferred resource for phage isolations which survive on drug-resistant bacteria (Du et al., 2021).

### 3.2 Optimal MOI and one-step growth assays

**Figure 2:**
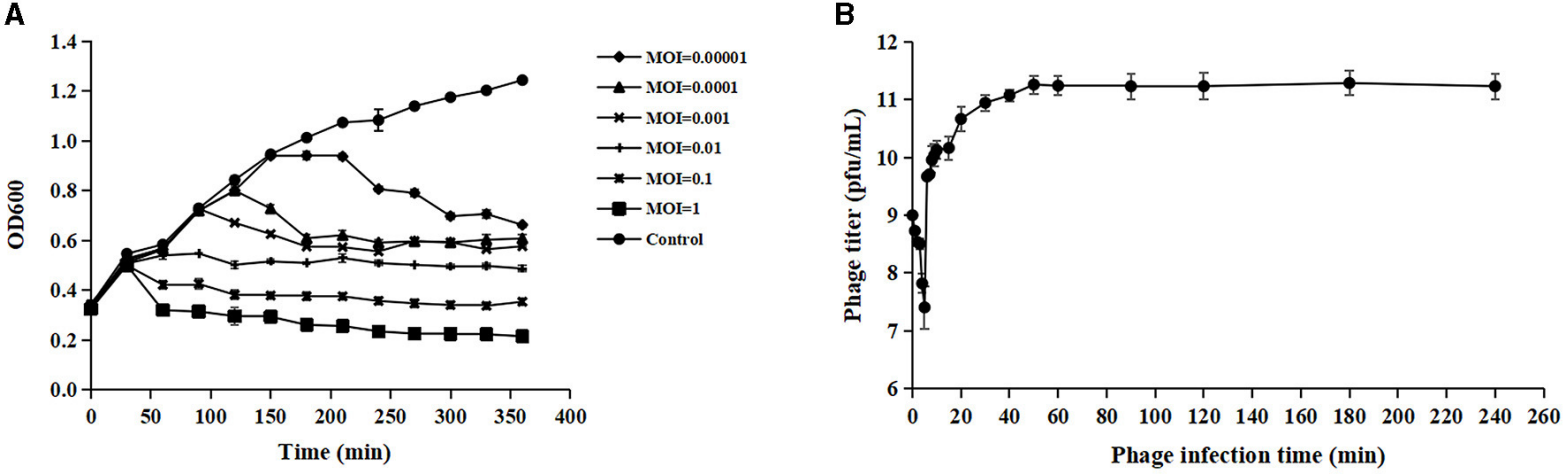
Biological characteristics of Acinetobacter virus Abgy202141. **(A)** Efficacy of Acinetobacter virus Abgy202141 infection at varying MOIs. **(B)** One-step growth curve of Acinetobacter virus Abgy202141. The data were obtained from three independent experiments and each mean shows bars as SD.

Phage adsorption to the host cell is the first step of infection, but not all adsorbed phages can successfully infect host cells (Hyman and Abedon, 2010). Acinetobacter virus Abgy202141 effectively reduced the growth of the host bacteria, and OD values declined more quickly at MOI = 1 than at other MOIs (0.1, 0.01, 0.001, 0.0001 or 0.00001) (Figure 2A). Hence the phage particles at an MOI of 1 were mixed with *A. baumannii* for one-step growth assays. The specific time points of one-step growth assays (1, 2, 3, 4, 5, 6, 7, 8, 9, 10, 15, 20, 30, 40, 50, 60, 90, 120, 180, and 240 min) were based on previously studies with slight modifications (Wu et al., 2018; Tang et al., 2019; Bozdeveci et al., 2021). In our research, the kinetics of adsorption indicated that about 65.3% of Acinetobacter virus Abgy202141 particles adsorbed to the surface of the *A. baumannii* cells within 2 min, and about 96.9% adsorbed within 5 min (Figure 2B). Efficient phage therapy is associated with phage adsorption, the yield of phages per infected cell (burst size), latency period, and initial dose (Weld et al., 2004). To determine the latency period and burst size of Acinetobacter virus Abgy202141, the one-step growth assay was performed. The latency period of Acinetobacter virus Abgy202141 was about 5 min, and the release of viral particles gradually increased within about 45 min (Figure 2B). The burst size of Acinetobacter virus Abgy202141 was about 189 phage particles per infected host cell similar to results of Yuan et al. (2021).

### 3.3 Stability of Acinetobacter virus Abgy202141

**Figure 3:**
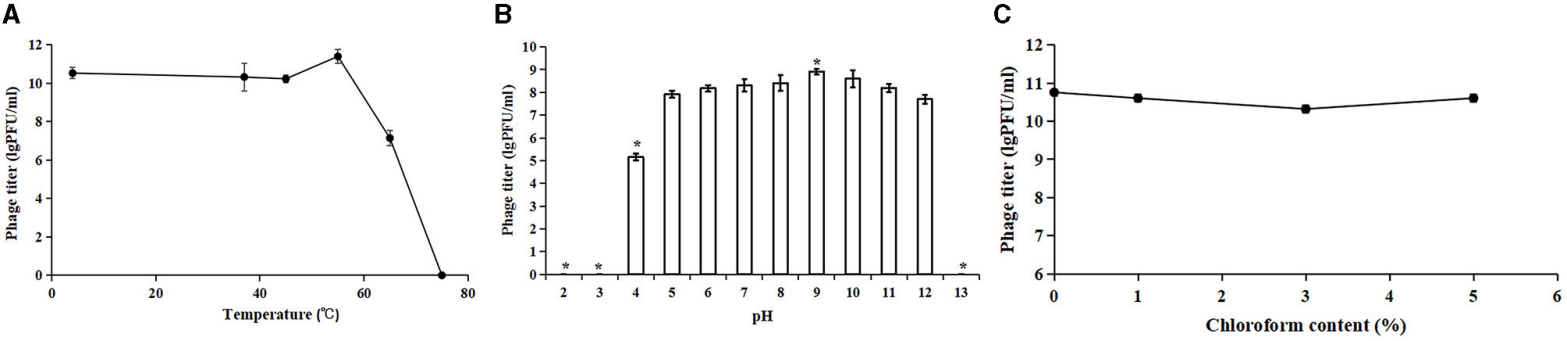
Stability of Acinetobacter virus Abgy202141. **(A)** Thermal stability of Acinetobacter virus Abgy202141. **(B)** Infectivity of Acinetobacter virus Abgy202141 tested over a range of pH; Significant differences are indicated by (^*^P < 0.05). **(C)** Chloroform sensitivity of Acinetobacter virus Abgy202141. All the experiments were repeated three times, and the data are shown as mean ± SD in the graphs.

To evaluate the potential therapeutic and other applications of Acinetobacter virus Abgy202141, the temperature and pH stability of Acinetobacter virus Abgy202141 were investigated. The titer of Acinetobacter virus Abgy202141 ranged from 1.8 × 10^10^ PFU/ml to 3.0 × 10^11^ PFU/ml and was only slightly affected by temperatures from 4°C to 55°C (Figure 3A). However, when the temperature reached 65°C, the titer of Acinetobacter virus Abgy202141 was rapidly decreased to 10^7^ PFU/ml. When the temperature reached 75°C, there was total loss of infectiveness. The results were similar to the reported phage AB1, where only 0.52% of phage AB1 survived after 60 min of incubation at 70°C (Yang et al., 2010). Acinetobacter virus Abgy202141 was treated with different pH values. After 1 h incubation, Acinetobacter virus Abgy202141 was found to be stable from pH = 4 to pH = 12 (Figure 3B). But no phage particles survived at pH < 4 or pH>12 (Figure 3B). These results indicated that extreme pH values may affect the stability of Acinetobacter virus Abgy202141, which was similar to that reported for phage AB1 (Yang et al., 2010). The chloroform tolerance of Abgy2021-4-1 was also investigated, and the results showed that the titer of Abgy2021-4-1 did not change significantly with different concentrations of chloroform from 0 to 5% (Figure 3C). Therefore, Abgy2021-4-1 might not contain lipids (Wei et al., 2023). These results were consistent with those of phage vB_EcoP_E212 (Wei et al., 2023).

Acinetobacter virus Abgy202141 showed broad tolerance toward chloroform concentration, pH values, and variations in thermal conditions, which should be considered beneficial for the storage and preparations of phage for potential application in clinical settings.

### 3.4 Identification of Acinetobacter virus Abgy202141 structural proteins

**Figure 4:**
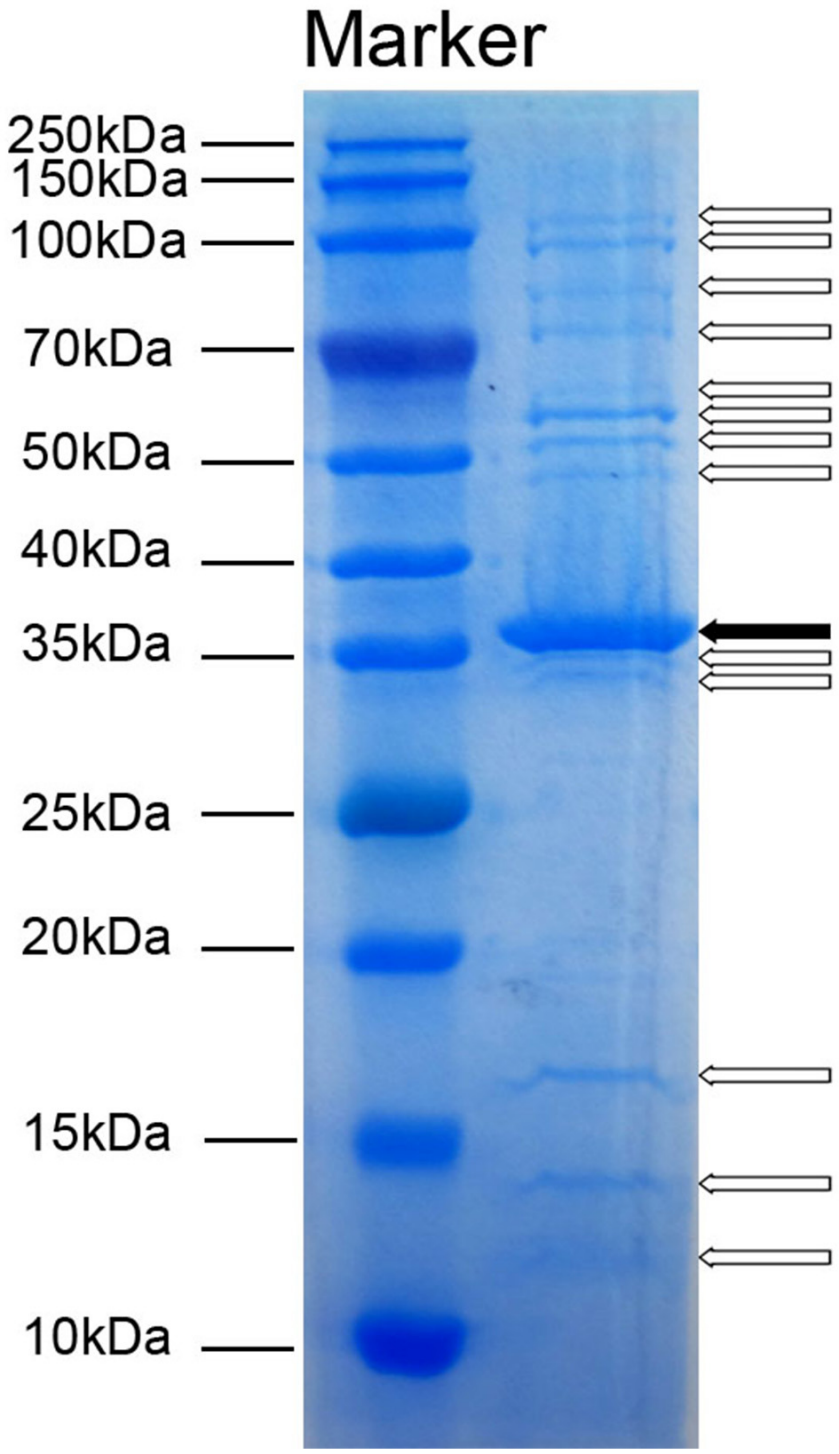
SDS-PAGE analysis of structural proteins in Acinetobacter virus Abgy202141. The molecular weights of the protein bands from 10 to 250 kDa are shown on the left. The solid arrow indicates a major protein band and blank arrows show minor protein bands.

To detect the number of structural proteins of Acinetobacter virus Abgy202141, we performed an SDS-PAGE analysis of purified Acinetobacter virus Abgy202141 particles. Fourteen protein bands ranging from 12 to 125 kDa, were observed on the gel (Figure 4). The 36 kDa protein was the most abundant, indicating that it was the major protein of Acinetobacter virus Abgy202141, and others were minor (Yang et al., 2010). These structural proteins were associated with phage assembly, regulation functions, sensing environmental stimulation sensing, and repair of tail fibers in retracted conformation (Yang et al., 2010; Cardarelli et al., 2011).

### 3.5 Annotation and analysis of the Acinetobacter virus Abgy202141 genome

**Figure 5:**
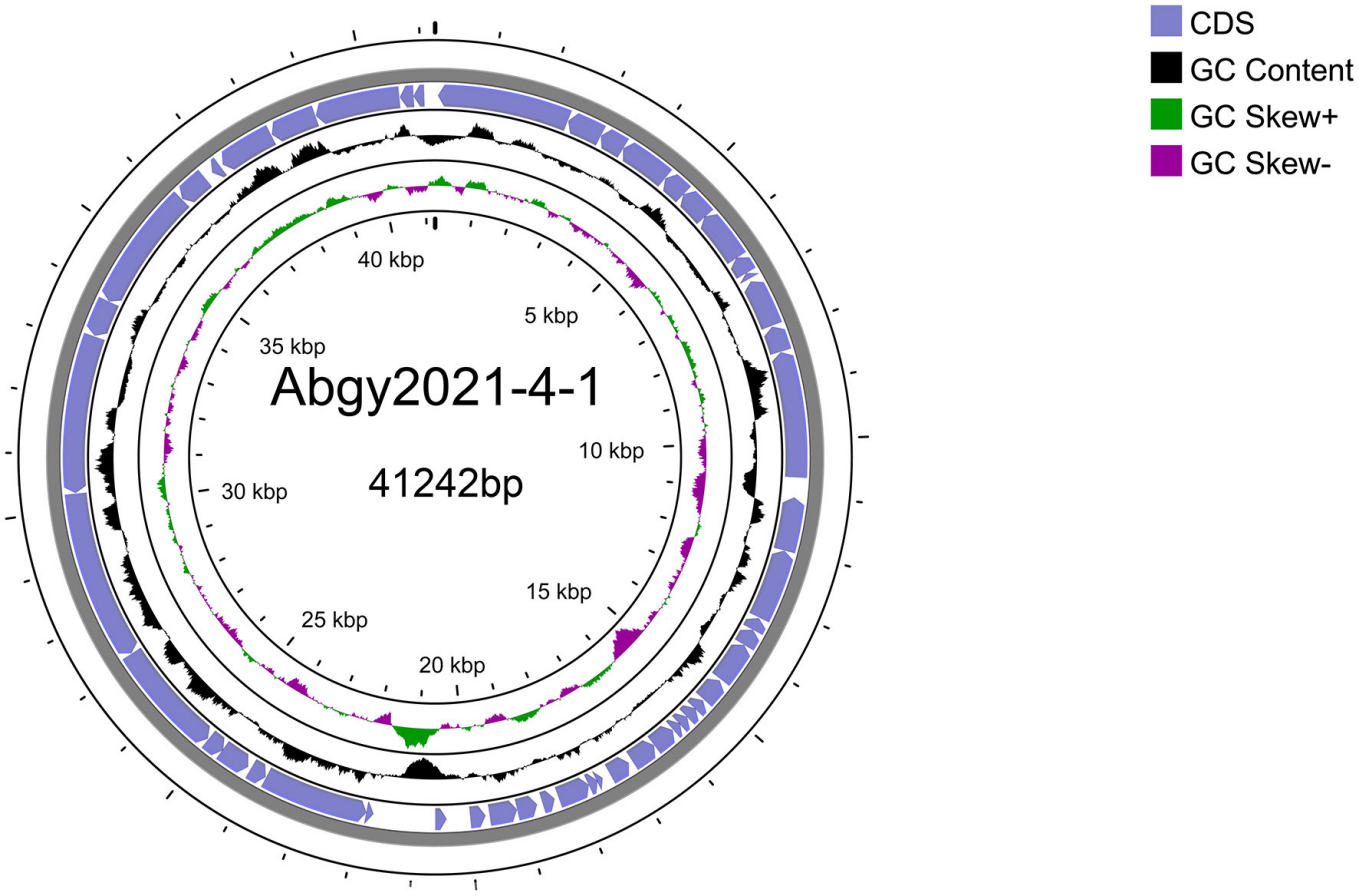
Schematic diagram of the genomic structure of Acinetobacter virus Abgy202141. The blue solid arrows indicate the reading direction of the encoding regions.

Acinetobacter virus Abgy202141 with double-stranded linear DNA as its genome is 41,242 bp long with 39.4% GC content (Figure 5). The complete genomic sequence of Acinetobacter virus Abgy202141 has been deposited in the GenBank database with the accession number OR770645. According to RAST and BLASTp analyses, a total of 50 ORFs were found in the Acinetobacter virus Abgy202141 genome, 29 of which had significant homology with known functional proteins, while the remaining genes were annotated as hypothetical proteins (Table 1). There were no recognized virulence or antimicrobial resistance genes in the Acinetobacter virus Abgy202141 genome (Table 1). No genes related to lysogenicity were found in the genome of Acinetobacter virus Abgy202141, which indicated Abgy202141 is a lytic phage (Merabishvili et al., 2014). Moreover, whole genome analysis of Abgy202141 with PhageAI also showed this phage is lytic (Tynecki et al., 2020).

**Table 1:** Predicted ORFs in the genome of Abgy202141.

**ORF**	**Nucleotide position**		**Gene Product**		**Putative function and best match**	**Matching GenBank Protein**	**Blastp result**		
	**Start**	**Stop**	**PI**	**MW (KDa)**			**Query cover (%)**	**Identity (%)**	***E*** **-value**
1	51	2468	6.44	90.84	RNA polymerase (Acinetobacter phage Abp1)	YP_008058226.1	100	99.50	0.00E+00
2	2477	3127	4.87	25.01	deoxynucleoside monophosphate kinase (Acinetobacter phage vB_AbaP_APK48-3)	QGH71555.1	100	95.83	2.00E-149
3	3129	3593	9.91	17.74	HNH endonuclease (Cronobacter sakazakii)	WP_076738056.1	60	50.00	3.00E-15
4	3593	4528	7.28	35.50	phosphoestherase with HTH domain (Acinetobacter phage AB_SZ6)	URQ05085.1	100	99.68	0.00E+00
5	4532	4972	9.77	16.61	endonuclease VII (Acinetobacter phage vB_AbaP_B1)	YP_009610315.1	100	100.00	4.00E-105
6	4969	5538	5.16	21.81	tRNA nucleotidyltransferase (Acinetobacter phage vB_Ab4_Hep4)	UVD33001.1	99	95.21	3.00E-130
7	5528	6481	5.64	35.95	5′-3′ exonuclease (Acinetobacter phage APK77)	UAW09898.1	100	99.68	0
8	6474	6806	8.93	13.16	hypothetical protein (Acinetobacter phage vB_AbaP_ABWU2101)	UFJ83450.1	100	100.00	4.00E-77
9	6803	6922	4.24	4.41	hypothetical protein (Acinetobacter phage Abp1)	YP_008058220.1	100	97.44	2.00E-19
10	6979	7869	5.14	32.38	5′-3′ exonuclease (Acinetobacter phage AbpL)	UVD42114.1	100	98.31	0
11	7887	8366	9.59	18.63	HNH endonuclease (Acinetobacter phage SWH-Ab-1)	YP_009949038.1	100	98.74	1.00E-114
12	8375	10675	5.69	87.38	DNA polymerase (Acinetobacter phage SWH-Ab-1)	YP_009949037.1	100	99.22	0
13	11041	12021	6.34	36.87	DNA ligase (Acinetobacter phage SWH-Ab-3)	YP_009949082.1	100	98.47	0
14	12024	13322	5.44	48.34	putative DNA helicase (Acinetobacter phage Abp1)	YP_008058212.1	100	99.77	0
15	13335	13571	6.88	8.87	hypothetical protein (Acinetobacter phage Abp1)	YP_008058211.1	100	100.00	1.00E-50
16	13571	13888	4.70	11.81	hypothetical protein (Acinetobacter phage Abp1)	YP_008058210.1	100	99.05	9.00E-69
17	13888	14643	8.72	28.89	DNA primase (Acinetobacter phage Abp1)	YP_008058209.1	100	99.60	0
18	14673	15122	9.49	16.90	HNH endonuclease (Acinetobacter phage Abp1)	YP_008058208.1	100	100.00	6.00E-107
19	15144	15353	8.98	7.86	hypothetical protein (Acinetobacter phage Abp1)	YP_008058207.1	100	100.00	1.00E-42
20	15343	15561	9.05	7.91	hypothetical protein (Acinetobacter phage Abp1)	YP_008058206.1	100	100.00	9.00E-44
21	15558	15749	9.83	7.12	hypothetical protein (Acinetobacter phage Abp1)	YP_008058205.1	100	100.00	1.00E-38
22	15736	15903	10.15	6.83	hypothetical protein (Acinetobacter phage vB_AbaP_PD-AB9)	YP_009189862.1	100	100.00	1.00E-29
23	15914	16348	9.32	16.29	hypothetical protein (Acinetobacter phage vB_AbaP_PD-AB9)	YP_009189863.1	100	100.00	2.00E-102
24	16350	16838	9.49	18.10	hypothetical protein (Acinetobacter phage Abp1)	YP_008058202.1	100	100.00	6.00E-116
25	16909	17307	6.39	14.80	hypothetical protein (Acinetobacter phage Abp1)	YP_008058201.1	100	99.24	2.00E-92
26	17466	17570	4.87	3.87	hypothetical protein (Acinetobacter phage Abp1)	YP_008058200.1	100	100.00	9.00E-16
27	17563	17742	9.86	6.58	hypothetical protein (Acinetobacter phage MRABP9)	WAK44726.1	100	100.00	1.00E-36
28	17739	18335	6.15	22.63	hypothetical protein (Acinetobacter phage Abp1)	YP_008058198.1	100	100.00	5.00E-147
29	18410	18643	6.57	9.32	hypothetical protein (Acinetobacter phage Abp1)	YP_008058197.1	100	100.00	2.00E-48
30	18744	19118	4.67	14.51	hypothetical protein (Acinetobacter phage Abp1)	YP_008058196.1	100	100.00	2.00E-86
31	19120	19629	6.52	18.59	hypothetical protein (Acinetobacter phage Abp1)	YP_008058195.1	100	98.82	1.00E-118
32	19702	19983	4.50	9.98	hypothetical protein (Acinetobacter phage Abp1)	YP_008058194.1	100	100.00	4.00E-60
33	20413	20619	9.30	7.98	hypothetical protein (Acinetobacter phage Abp1)	YP_008058193.1	100	100.00	6.00E-43
34	21747	21881	8.10	5.13	DNA binding protein (Acinetobacter phage IME-200)	YP_009216494.1	100	100.00	1.00E-21
35	21878	23815	6.54	72.86	terminase large subunit (Acinetobacter phage Abp1)	YP_008058244.1	100	99.84	0
36	23825	24133	4.40	11.19	DNA maturase A (Acinetobacter phage vB_AbaP_APK14)	AYR04397.1	100	99.02	2.00E-65
37	24193	24750	9.48	21.01	putative endolysin (Acinetobacter phage vB_AbaP_PMK34)	QGF20176.1	100	99.46	1.00E-130
38	24737	25072	5.70	11.94	holin/anti-holin (Acinetobacter phage SH-Ab 15519)	YP_009598267.1	100	100.00	4.00E-73
39	25086	27167	5.10	75.35	tail fiber protein (Acinetobacter phage SH-Ab 15519)	YP_009598268.1	100	99.57	0
40	27174	30272	5.88	113.96	internal virion protein with endolysin domain (Acinetobacter phage Abp1)	YP_008058238.1	100	99.71	0
41	30282	33167	7.66	105.76	internal virion protein B (Acinetobacter virus fBenAci001)	QOV07746.1	100	98.75	0
42	33180	33854	8.80	23.51	internal virion protein A (Acinetobacter phage vB_AbaP_APK2)	AZU99239.1	100	98.21	3.00E-157
43	33854	36145	5.00	84.35	tail protein (Acinetobacter phage phiAB1)	YP_009189376.1	100	98.69	0
44	36154	36714	9.46	21.59	tail tabular protein A (Acinetobacter phage vB_AbaP_PMK34)	QGF20169.1	100	98.92	6.00E-132
45	36878	37063	4.71	6.43	tail protein (Acinetobacter phage AB3)	YP_008060142.1	100	100.00	5.00E-33
46	37119	38150	5.41	38.36	capsid and scaffold protein (Acinetobacter phage vB_AbaP_ZHSHW)	UPT53553.1	100	100.00	0
47	38166	39026	5.38	30.51	head scaffolding protein (Acinetobacter phage vB_AbaP_B5)	YP_009610424.1	100	98.60	0
48	39035	40591	4.97	58.89	head-tail adaptor (Acinetobacter phage vB_AbaP_AS11)	YP_009599271.1	100	100.00	0
49	40600	40851	6.21	9.04	hypothetical protein (Acinetobacter phage Abp1)	YP_008058228.1	100	100.00	4.00E-53
50	40848	41045	5.25	7.69	hypothetical protein (Acinetobacter phage Abp1)	YP_008058227.1	100	100.00	8.00E-40

**Figure 6:**
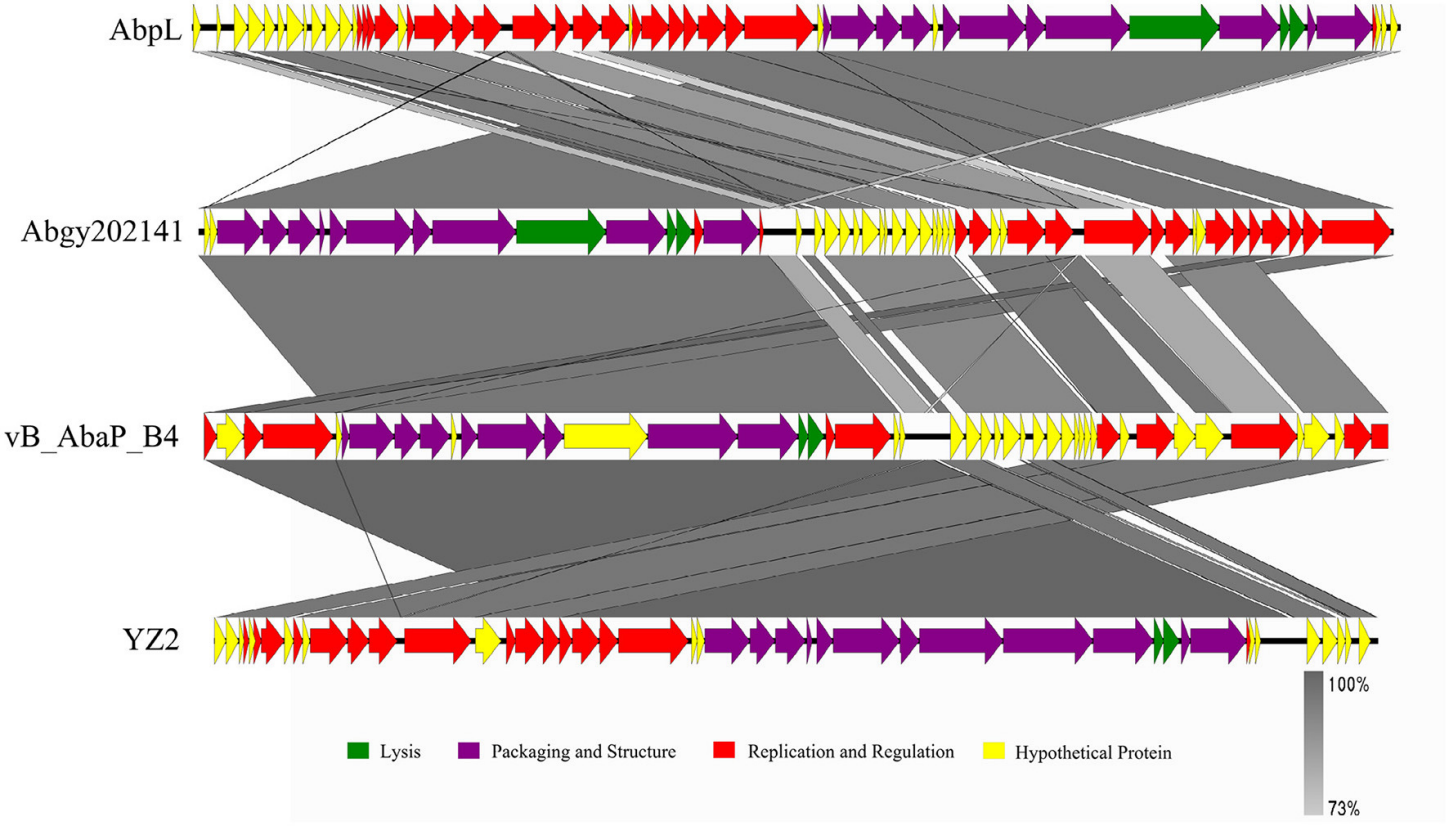
Comparison of the genome of Acinetobacter virus Abgy202141 (second) to Acinetobacter phage AbpL (top), Acinetobacter phage vB_AbaP_B4 (third), and Acinetobacter phage YZ2 (bottom). The different color arrows indicate the CDS in the full length of the genomic sequence. The transcript direction of each CDS is indicated by the direction of the arrow. Homology is represented by the slanted gray bars, and the degree of sequence similarity is indicated by the grayness intensity.

In order to determine the genomic similarity between Acinetobacter virus Abgy202141 and other phages, BLASTn of the complete genome was done against the NCBI database, and three phages were found with the most similarity to Acinetobacter virus Abgy202141. The nucleotide similarity between Acinetobacter phage AbpL and Acinetobacter virus Abgy202141 was 95.23% over their entire lengths, while the nucleotide similarity with Acinetobacter phage vB_AbaP_B4 and Acinetobacter phage YZ2 were 95.12% and was 95.05%, respectively. Detailed information on the three matching phages is shown in Table 2. The modular structure of the genome was compared among the three phages, and this mainly involved several functional groups associated with morphogenesis and structure, DNA replication, repair, recombination and processing, biological metabolism, transcription, lysis, assembly and packaging, and a HNH endonuclease (Figure 6). The distribution of genes with the same function varied among the different phages, and this implied that Acinetobacter virus Abgy202141 is a novel phage.

**Table 2:** Information on the three closest phage strains to Abgy202141.

**Acinetobacter phage**	**Accession Number**	**Host**	**Location**	**Query cover against Abgy202141**	**Identity against Abgy202141**
Acinetobacter phage AbpL	OP171942	*A. baumannii*	China	91%	95.23%
Acinetobacter phage vB_AbaP_B4	OR584314	*A. baumannii*	China	93%	95.12%
Acinetobacter phage YZ2	OR660046	*A. baumannii*	China	89%	95.05%

There were three proteins putative endolysin (ORF37), holin/anti-holin (ORF38), and internal virion protein with endolysin domain (ORF40), which were involved in host lysis. Holin could form non-specific channels or holes on the cytoplasmic membrane of bacteria, which allow endolysins to escape and lyse the peptidoglycan (Wang et al., 2000; Young, 2014). The tail fiber protein, which was homologous to ORF39 in the Abgy202141 genome, is believed to be involved in the process of tail assembly or penetration of the host cell outer membrane after infection (Nobrega et al., 2018). The tail fiber protein (ORF39), tail protein (ORF43), tail tabular protein A (ORF44), tail protein (ORF45) and head-tail adaptor (ORF48) are likely responsible for the host range of the phage (Nobrega et al., 2018). The capsid and scaffold protein (ORF46) not only protects the phage genome from degradation in harsh extracellular environments but also maintains stability against high internal pressure during the DNA packaging process (Rao and Black, 2010). The ORF35 is predicted to be the terminase large subunit, which functions in ATP binding, precursor binding, and DNA cleavage (Sun et al., 2010). ORF41 encodes the internal virion protein B. ORF42 encodes the internal virion protein A. ORF47 is predicted to be the head scaffolding protein. ORF1 is predicted to be the RNA polymerase. RNA polymerase participates in gene expression and can transcribe DNA into RNA (Shin and Murakami, 2021). ORF2 is predicted to be the deoxynucleoside monophosphate kinase. This enzyme is involved in the biosynthesis of deoxyribonucleotides (Bao and Ryu, 2006). ORF4 is predicted to be the phosphoestherase with HTH domain. It participates in DNA repair and replication (Aravind et al., 2005). ORF5 is predicted to be the endonuclease VII. It is a component of the mismatch repair mechanism in the genome (Shcherbakov et al., 2011). ORF7 and ORF10 are predicted to be the 5'-3' exonuclease. This enzyme can promote gap to translate into DNA in DNA synthesis reactions (Longley et al., 1990). ORF12 is predicted to be the DNA polymerase. It plays an important role in DNA replication and repair (Ghosh and Raghavan, 2021). ORF13 is predicted to be the DNA ligase. It can catalyze the formation of phosphodiester bonds and is crucial for DNA repair and cell replication (Caracciolo et al., 2022; Duckworth et al., 2023). ORF14 is predicted to be the putative DNA helicase. DNA helicase and DNA polymerase work together to release parental DNA and maintain chromosome stability (Wu and Brosh, 2012; Lo and Gao, 2021). ORF17 is predicted to be the DNA primase. It facilitates chromosome replication and repair, which is crucial for maintaining the stability of telomeres and chromosome (Arezi and Kuchta, 2000). ORF3, ORF11 and ORF18 encode the HNH endonuclease, that is also involved in DNA packaging (Kala et al., 2014). ORF34 is predicted to be the DNA binding protein. It plays important roles in DNA replication, DNA packaging, and DNA repair (Manavi et al., 2023). ORF36 is predicted to be the DNA maturase A. Moreover, no virulence-related genes, antibiotic-resistance genes, or tRNAs were found.

**Figure 7:**
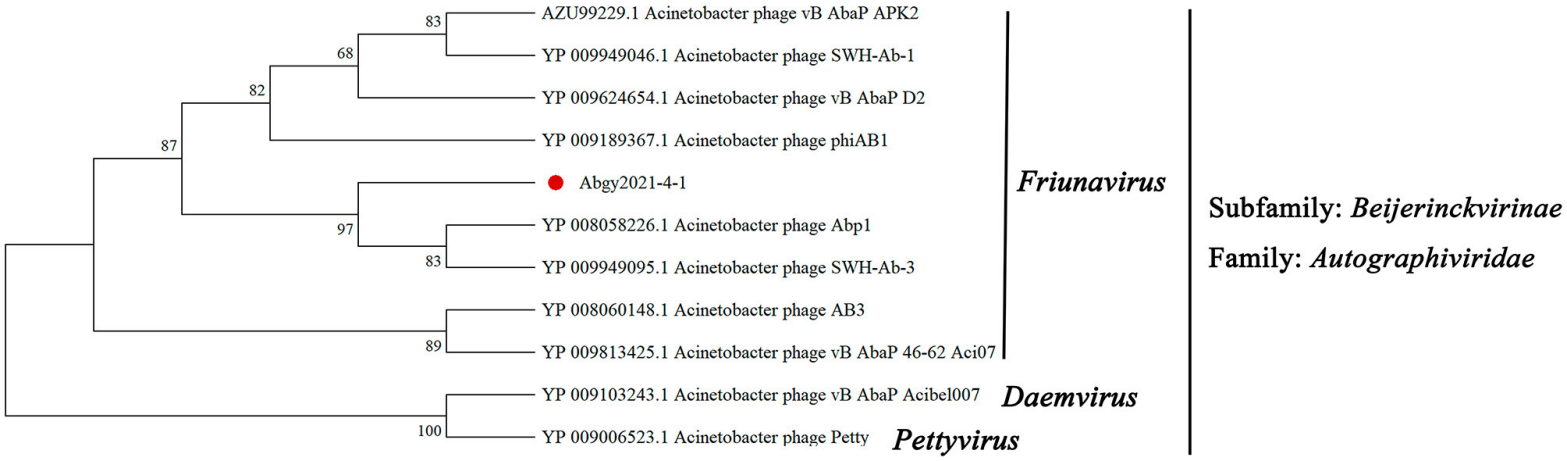
The Maximum Likelihood tree was constructed using RNA polymerase of Acinetobacter virus Abgy202141 and related phages. The amino acid sequences of the related phages were obtained from NCBI, and the accession numbers are shown in the figure. The red dot indicates the position of Acinetobacter virus Abgy202141. Bootstrap percentages out of 1000 replications are shown on the branches.

To further investigate the taxonomic status of Acinetobacter virus Abgy202141, a phylogenetic tree was constructed with RNA polymerase from various phages following (Merabishvili et al., 2014). The results were divided into two branches. Acinetobacter virus Abgy202141 had a closer relationship with phages such as Acinetobacter phage SWH-Ab-3 and Acinetobacter phage Abp1 of the genus *Friunavirus*, while there was a more obvious distinction with phages of the genus *Daemvirus* and *Pettyvirus*. The results showed that Acinetobacter virus Abgy202141 belonged to the genus *Friunavirus* in the *Autographiviridae* family (Figure 7).

### 3.6 *In vivo* synergy in the *G. mellonella* model

**Figure 8:**
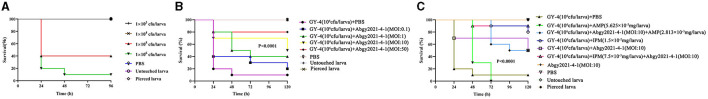
Therapeutic evaluation of Acinetobacter virus Abgy202141 against *A. baumannii* injected into *G. mellonella*. **(A)** Survival of *G. mellonella* larvae infected with different concentrations of *A. baumannii* strain GY-4. **(B)** Survival rates after application of Acinetobacter virus Abgy202141 for treating *A. baumanii* strain GY-4 infection in *G. mellonella* larvae. **(C)** Survival rates after treatment with Acinetobacter virus Abgy202141 phage combined with antibiotics for *A. baumannii* GY-4 infection in *G. mellonella* larvae.

*G. mellonella* larvae were used as an animal model to test the ability of Acinetobacter virus Abgy202141 to eliminate *A. baumannii* strain GY-4. The different doses (1 × 10^3^ CFU/larva, 1 × 10^4^ CFU/larva, 1 × 10^5^ CFU/larva, 1 × 10^6^ CFU/larva) were injected into *G. mellonella* larvae. We found 10^6^ CFU/larva led to a constant decrease of larval survival within 96 h (Figure 8A), and was chosen as the optimal concentration to look for some possible differences in the pathogenicity of *A. baumannii*. Twenty minutes after injection (10^6^ CFU/larva) of *A. baumannii* strain GY-4, the purified Acinetobacter virus Abgy202141 at different MOIs (50, 10, 1, and 0.1) were injected into the inoculated *G. Mellonella* larvae. We found that the survival of larvae increased constantly with increasing MOIs of Acinetobacter virus Abgy202141 (Figure 8B). When the injection level of Acinetobacter virus Abgy202141 reached an MOI of 50, the survival of *G. Mellonella* larvae was up to 80 % (Figure 8B). The results of the *G. mellonella* larvae model test indicated a higher threshold *in vivo*, which reinforced the need for thorough evaluation prior to any clinical application. Beside mice models, there are still other simple *in vivo* tests (Manohar et al., 2022). However, the animal model involving *G. mellonella* is a common infection model used to evaluate phage efficacy (Tsai et al., 2016). Although insect larvae do not have an adaptive immune system, they can be used as a valuable model to evaluate the therapeutic value of a phage and the potential ability to clear the pathogen in the more complex environment of larger animals (Zhang et al., 2022). The results showed that Abgy202141 was highly efficient in clearing a large quantity of bacteria under our experimental conditions. However, to use the Abgy202141 in clinical practice, a mouse model should be first tested to evaluate phage efficacy.

Antibiotics combined with phage had previously been investigated *in vitro* and in animal models (Segall et al., 2019). So, antibiotics combined with phage were also used for treating GY-4 infection in *G. mellonella* larvae in this study. The survival rates of larvae were 0, 80% and 50% within 120 h, after treatment with AMP, IPM, or phage respectively (Figure 8C). But the survival rate of larvae after 120 h was 90%, when treated with the combination of IPM and phage (Figure 8C). In addition, no death or blackening of non-inoculated larvae were observed after injection of either the phage alone or PBS buffer as negative controls. Compared with PBS and AMP treatment, Acinetobacter virus Abgy202141 treatment significantly improved the survival rate of inoculated larvae. The survival rate from the IPM treatment was higher than the Acinetobacter virus Abgy202141 treatment. However, the combination of IPM and Acinetobacter virus Abgy202141 gave the highest survival rate. The potential of phage-antibiotic synergy for treatment of MDR bacterial infections is gaining greater attention (Segall et al., 2019). In another study, a T4-like phage, KARL-1, could infect eight MDR strains of *A. baumannii*, and it showed significant synergy with meropenem, and modest synergy with ciprofloxacin or colistin (Jansen et al., 2018). A study of mechanisms of phage-antibiotic synergy have been investigated between phage and antibiotics against either planktonic (vegetative) or biofilm-growing methicillin-resistant *Staphylococcus aureus* (MRSA) (Tkhilaishvili et al., 2018). Phage SB-1 degraded the MRSA polysaccharide matrix, and targeted persister cells, which made SB-1 combined with any of the five antibiotics assayed (rifampin, daptomycin, fosfomycin, ciprofloxacin, or vancomycin) even more effective (Tkhilaishvili et al., 2018). Hence the mechanisms of Abgy202141 combined with antibiotics synergy should be investigated in further study.

Phage therapy has become popularized worldwide (Uyttebroek et al., 2022). However, phage therapy has limitations including variation among clinical pathogens in resistance to phages, and host immune response to phage (Hatfull et al., 2022). Therefore, with these considerations, phage therapy is commonly used as a last resort in response to complete antibiotic failure (Hatfull et al., 2022), and a major advantage of phage therapy is treatment of MDR bacteria (Zalewska-Piatek, 2023). Moreover, the phages utilized in combination with antibiotics have been associated with successful microbiological and clinical responses, when traditional antimicrobial therapy have failed (Schooley et al., 2017; Chan et al., 2018; Hatfull et al., 2022).

## 4 Conclusions

In this study, Acinetobacter virus Abgy202141 was isolated from underground sewage with *A. baumannii* strain GY-4 as the bait. Stability of Acinetobacter virus Abgy202141 showed broad tolerance to chloroform, various pH conditions, and thermal stability. Based on genomic analysis and morphological characteristics, Acinetobacter virus Abgy202141 belongs to the genus *Friunavirus* in the *Autographiviridae* family. Acinetobacter virus Abgy202141 might be used as an alternative for antibiotics, because its genome did not carry any virulence-related or antibiotic-resistance genes. Results using the insect model (*G. mellonella*) assay also showed the therapeutic potential of Acinetobacter virus Abgy202141 combined with IPM. However, the application of Abgy202141 for clinical practice needs further study, such as in a mouse model to evaluate phage efficacy.

## Data availability statement

The datasets presented in this study can be found in online repositories. The names of the repository/repositories and accession number(s) can be found in the article/supplementary material.

## Author contributions

XT: Formal analysis, Investigation, Methodology, Validation, Writing – original draft. XL: Formal analysis, Investigation, Methodology, Validation, Writing – review & editing. JZ: Data curation, Software, Writing – review & editing. LW: Resources, Writing – review & editing. QW: Methodology, Writing – review & editing. XQ: Methodology, Writing – review & editing. JL: Methodology, Writing – review & editing. DZ: Resources, Writing – review & editing. TH: Methodology, Writing – review & editing. YJ: Conceptualization, Formal analysis, Funding acquisition, Methodology, Resources, Writing – original draft, Writing – review & editing.
